# Serum and salivary tissue transglutaminase IGA (tTG-IGA) level in celiac patients

**DOI:** 10.1186/s12876-022-02456-x

**Published:** 2022-08-06

**Authors:** Mehran Ajdani, Nazanin Mortazavi, Sima Besharat, Saeed Mohammadi, Taghi Amiriani, Ahmad Sohrabi, Alireza Norouzi, Ghezeljeh Edris

**Affiliations:** 1 Dental Research Center, Golestan University of Medical Sciences, Gorgan, Iran; 2 Department of Oral and Maxillofacial Medicine, School of Dentistry, Golestan University of Medical Sciences, Gorgan, Iran P.O. Box 4916953363,; 3 Golestan Research Center of Gastroenterology and Hepatology, Golestan University of Medical Sciences, Gorgan, Iran; 4 Stem Cell Research Center, Golestan University of Medical Sciences, Gorgan, Iran; 5 Cancer Control Research Center, Cancer Control Foundation, Iran University of Medical Sciences, Tehran, Iran

**Keywords:** Celiac disease, Serologic test, Saliva, Xerostomia, Interleukins

## Abstract

**Background:**

Celiac disease (CD) is a genetically determined autoimmune disease triggered by gluten consumption. Patients with these conditions have intraepithelial lymphocytosis, crypt hyperplasia, and severe intestinal atrophy. Gluten elimination is the only way to reduce this chronic inflammation. The diagnosis of CD is usually made by analyzing anti-tTG, anti-DGP, or EMA serological tests, and it is confirmed by biopsy of the duodenum.

In people with CD, xerostomia or dry mouth is a common complication. This condition causes the salivary glands to malfunction and, in turn, may result in oral plaque and periodontal disease.

By comparing salivary and serum levels of tissue transglutaminase IgA (tTG-IgA), this study aims to suggest a non-invasive method for diagnosis of CD. Furthermore, the present study evaluates the severity of xerostomia symptoms in people with CD.

**Methods:**

In this case–control study, participants were patients referred to the internal ward of Sayyad Shirazi hospital. The control group was selected from healthy people who attended Gorgan Dental College.

In this study, an analysis of serum was performed following consent from patients. This was followed by a salivary test, and the results of both tests were compared.

The Xerostomia Inventory questionnaire was also used to determine the severity of xerostomia. As part of this study, examination of factors such as total protein concentration of saliva, albumin concentration, amylase level, pH, sodium, calcium, potassium, phosphorus, and interleukin (6, 18, and 21) were conducted.

**Results:**

A total of 78 people were studied (aged 15 to 68), 26 were male (33.3%) and 52 were female (66.7%). In comparisons of the serum and saliva of people with and without CD, the level of amylase was higher in the latter group. The average levels of IL-6، IL-18 ،IL-21, and salivary and serum tTG were higher in people with CD. Additionally, CD patients were more likely to develop xerostomia.

**Conclusion:**

Study findings showed that CD can reduce certain salivary enzymes and elements, as well as increase inflammatory cytokines, salivary, and serum tTG.

The management of dry mouth should also be recommended for celiac disease patients in order to prevent its complications.

## Introduction

Celiac disease (CD) is an autoimmune disease caused by ingestion of wheat gluten and related prolamines of barley and rye [1]. A characteristic feature of CD is the infiltration of inflammatory cells into the lamina propria, crypt hyperplasia, and villous atrophy, and CD is the leading cause of villous atrophy [2, 3].

It is estimated that the pooled global seroprevalence of CD in 2018 is 1.4%, while the pooled global prevalence of biopsy-confirmed CD is 0.7%, with the highest prevalence being in Europe (0.8%) and Oceania (0.8%), and the lowest in South America (0.4%) [4]. For studies that used serological methods for diagnosis, CD prevalence in Iran was estimated at 3% (95% CI 0.03–0.03) and 2% (95% CI 0.01–2.0%), for those that used biopsy methods [5].

Despite increasing awareness of the disease, the CD is believed to be underdiagnosed [6]. The preferred serologic test for detecting CD is IgA anti-tissue transglutaminase antibody (tTG IgA), which delivers high sensitivity (93%) and specificity (95%) [3]. Another test commonly used is IgA anti-endomysial antibody (EMA) which is the most specific of all assays, along with IgG-based test antibodies like anti-deaminated gliadin peptide or tTG antibodies for IgA deficient individuals [3]. tTG is a calcium-dependent enzyme that catalyzes the formation of ε‐(γ‐glutamyl) lysine bonds between glutamine and lysine residues in body fluids and tissues. Gliadin is a substrate for tTg, which deaminates the glutamine residues in gliadin, causing gut-derived T cells to recognize gliadin and activate an immune response [7]. The gold standard for the diagnosis of CD is antibody testing followed by a biopsy of the duodenum [8].

In addition to plasma, autoantibodies produced in CD by ingestion of gluten-containing foods also appear in saliva, but in smaller concentrations than in blood [9]. Although the tTG IgA concentration in human saliva is smaller than in blood, it is a noninvasive technique that bypasses the unpleasant process of collecting blood samples. Human recombinant tTG in a fluid-phase radioimmunoassay (RIA) format was most sensitive in detecting tTG-Abs [2]. Saliva versus serum RIA has a specificity of 100%, whereas the sensitivity is 97.4% [10]. An enzyme-linked immunomagnetic electrochemical (ELIME) assay was also developed for measuring serum TGA in saliva [11].

The present study aimed to investigate the correlation between salivary and serum tTG-Ab.

## Methods

### Study design

The present case–control study was conducted in Gorgan city, northeast of Iran. Subjects were patients that were referred to the internal ward of Sayyad Shirazi hospital. By gender matching, the control group was selected from healthy people referred to Gorgan Dental College. Subjects in the case group were on a gluten-free diet (GFD). The ethics committee of Golestan University of Medical Sciences approved the protocol.

#### Definition

The European Society of Pediatric Gastroenterology, Hepatology and Nutrition (ESPGHAN) guidelines and AGA Clinical Practice Update on Diagnosis and Monitoring of Celiac Disease suggest diagnostic criteria for CD. The guidelines state it depends upon gluten-dependent CD symptoms (particularly malabsorption), CD-specific antibody levels (high serum TGA IgA levels ≥ 10 × ULN) as criteria for CD diagnosis without a biopsy. Also, in the absence of a biopsy, the CD diagnosis should be confirmed with a positive EMA-IgA test in a second blood sample. Villous atrophy and crypt hyperplasia observed in a biopsy of the duodenum are histopathologic findings associated with HLA-DQ2 and/or HLA-DQ8. This study used ESPGHAN as an inclusion criterion for CD diagnosis in pediatrics, and AGA Clinical Practice Update to diagnose CD in adults [12, 13]. Patients with denture use, xerostomia due to another systemic disease, and selective IgA deficiency were excluded from the study.

The Xerostomia Inventory (XI) questionnaire was also used to evaluate the severity of xerostomia.

### Procedure

Informed consent was obtained from subjects before collecting serum and saliva samples. Following receipt of serum samples, the samples were stored at − 20 °C until analysis. During the clinical examination, the resting whole saliva was collected by the spit method. During the morning hours of 8 a.m. to 10 a.m., samples of saliva were collected into chilled, graduated glass tubes. Prior to sampling, donors were asked to refrain from eating, smoking, drinking (except water), using a mouth rinse, and brushing their teeth for at least one-hour Participants were instructed to spit into a plastic tube to collect saliva samples. The process took ten minutes to complete. A set of collected samples was placed on ice, and the samples were spun at 40 °C for 10 min at 10,000 rpm within two hours [10].

Subjects were also asked to complete an international validated XI questionnaire designed for quantifying the severity of dry mouth symptoms [14]. The XI contains 11 items on a 5-point Likert scale that describe the severity of mouth dryness on a scale ranging from 11 to 55. There are five options per response item: “Never”, “Hardly ever”, “Occasionally”, “Frequently”, and “Always” [15].

#### Biochemical measurement

Based on the manufacturer’s protocol, salivary and serum tTg-IgA antibody enzymes and inflammatory cytokines (IL-6, IL-18, and IL-21) were measured by ELISA.

Furthermore, physical and chemical factors such as total protein concentration in saliva, amylase level, albumin concentration, pH, sodium, calcium, potassium, and phosphorus were evaluated.

#### Statistical analysis

Quantitative data were described using mean and standard deviation and qualitative data were described based on the frequency and relative frequency. The Kolmogorov–Smirnov test was used to determine whether the data were normally distributed. Assuming the data were normal, the homogeneity of variance was determined using Leven's test. The mean values of the studied variables were compared based on independent t-tests in the homogeneity of variance situation. The Welch t-test is applied if the variances are not homogeneous. A Mann–Whitney U test was used if there were any abnormalities. Comparing the means of quantitative variables if data differences are normal was conducted using the paired t-test and otherwise the Wilcoxon signed-rank test. Comparisons of qualitative data were conducted using the Chi-square test or Fisher's exact test.

The XI questionnaire resulted in a Cronbach's alpha coefficient of 0.87, indicating reliable internal consistency. The questionnaire was evaluated in two groups of case and control participants.

In receiver operating characteristic (ROC) analysis, the area under the curve (AUC) was used to estimate the predictive power of salivary indicators. Sensitivity, specificity, positive predictive value (PPV), negative predictive value (NPV), and false discovery rate (FDR) were calculated.

All tests were two-sided, and P < 0.05 was regarded as statistically significant. Statistical analyses were conducted using R (v4.1.0).

## Results

A total of 78 individuals aged 15 to 68 participated in the study. The mean and SD of age in the case and control groups were 39.18 ± 11.26 and 34.31 ± 7.23, respectively. The overall gender distribution was 26 (33.7%) male (15 cases, 11 controls) and 52 female (24 cases, 28 controls). A total of 14 patients has experienced disease under five years and 25 have been affected for over five years.

Xerostomia assessment showed statistically significant differences between case and control groups. The levels of xerostomia were significantly higher in CD patients (2.44 ± 0.71) than in healthy control subjects (1.91 ± 0.69) (p-value = 0.001). The results of the XI questionnaire are presented in Table 1.

**Table 1:** Frequency distribution of Xerostomia Inventory questionnaire items

Items	Never number (%)	Hardly ever number (%)	Occasionally number (%)	Frequently number (%)	Always number (%)
I sip liquids to aid in swallowing food	25 (32.1)	19 (24.4)	17 (21.8)	11 (14.1)	6 (7.7)
My mouth feels dry when eating a meal	35 (44.9)	19 (24.4)	19 (24.4)	3 (3.8)	2 (2.6)
I get up at night to drink	32 (41)	10 (12.8)	33 (42.3)	3 (3.8)	0 (0)
My mouth feels dry	25 (32.1)	18 (23.1)	24 (30.8)	7 (9)	4 (5.1)
I have difficulty in eating dry foods	20 (25.6)	19 (24.4)	23 (29.5)	12 (15.4)	4 (5.1)
I suck sweets or cough lollies to relieve dry mouth	61 (78.2)	8 (10.3)	8 (10.3)	1 (1.3)	0 (0)
I have difficulties swallowing certain foods	32 (41)	19 (24.4)	19 (24.4)	6 (7.7)	2 (2.6)
The skin of my face feels dry	28 (35.9)	10 (12.8)	25 (32.1)	8 (10.3)	7 (9)
My eyes feel dry	38 (48.7)	12 (15.4)	20 (25.6)	7 (9)	1 (1.3)
My lips feel dry	22 (28.2)	7 (9)	28 (35.9)	14 (17.9)	7 (9)
The inside of my nose feels dry	33 (42.3)	11 (14.1)	27 (34.6)	5 (6.4)	2 (2.6)

The mean salivary pH, calcium, phosphorus, sodium, potassium, albumin, and total protein difference between the case and control groups were not statistically significant. The mean amylase in the case group was significantly lower than that in the control group. The mean levels of IL-18, IL-6, and IL-21 in the case group were significantly higher than those in the control group as shown in Table 2.

**Table 2:** Evaluation of salivary variables in study groups

	CD patients (Mean ± SD)	Control group (Mean ± SD)	p-value
Ph	7.78 ± 0.098	7.76 ± 0.095	0.478
Amylase	198.22 ± 84.69	812.20 ± 1260.26	< 0.001
Calcium	2.69 ± 2.12	2.16 ± 0.848	0.655
Phosphorous	17.76 ± 8.15	17.50 ± 5.03	0.764
Albumin	116.52 ± 72.26	105.85 ± 60.17	0.567
Total protein	67.44 ± 29.24	55.44 ± 15.24	0.07
Sodium	7.92 ± 4.068	6.40 ± 2.50	0.393
Potassium	23.72 ± 6.74	22.78 ± 3.002	0.530
IL-18	303.28 ± 202.48	166.20 ± 105.64	< 0.001
IL-6	9.38 ± 4.94	4.93 ± 2.24	< 0.001
IL-21	438.96 ± 194.68	339.59 ± 126.58	0.021
Salivary tTG	1822.37 ± 2232.81	95.04 ± 183.20	< 0.001

**Fig. 1:**
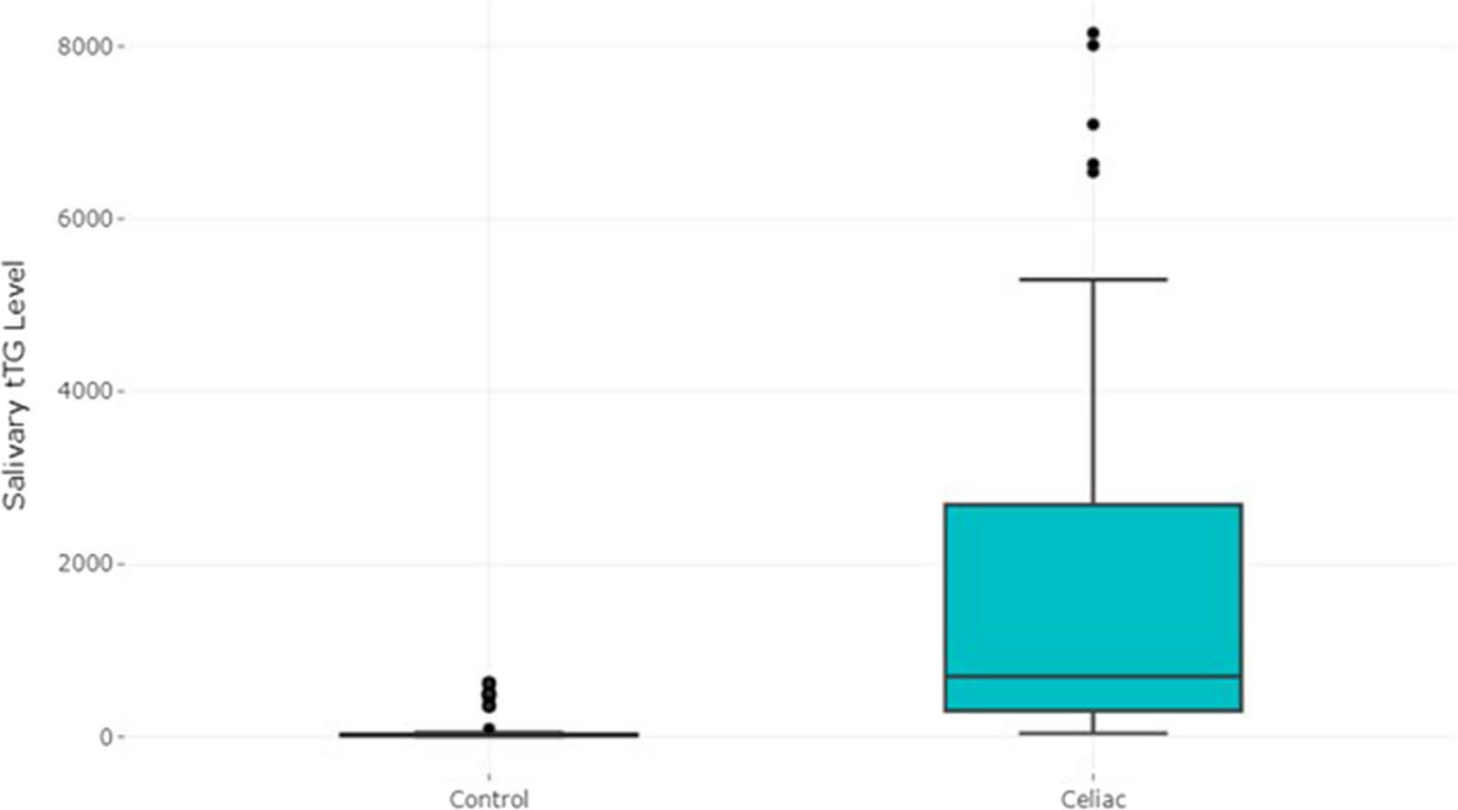
Box plot of salivary tTG in study groups

Mean salivary and serum tTG in the case group (1822.37 ± 2232.81 and 152.72 ± 47.57), compared with salivary and serum tTG in the control group (95.04 ± 183.20 and 3.58 ± 2.92), were significantly higher. Boxplot distributions for salivary tTG in case and control groups can be seen in Table 2 and Fig. 1. The plot shows that salivary tTG levels in case groups are significantly higher than in control groups.

**Fig. 2:**
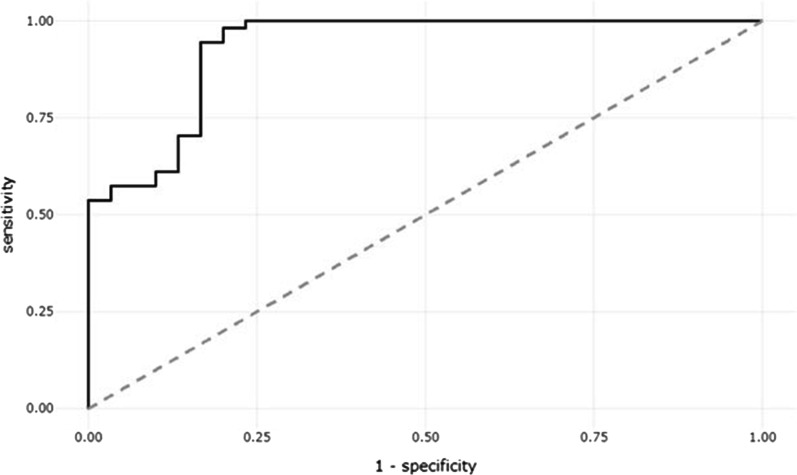
ROC curve of salivary tTG for diagnosis of CD

ROC curve analysis was used to determine the diagnostic value of salivary tTG-IgA. Considering serum tTG as the gold standard, salivary tTG test showed an AUC, sensitivity, specificity, and diagnostic accuracy of 0.9309 (95% CI = 0.8721–0.9896), 98.15%, 80.00%, and 91.67% respectively with a cutoff of 67.97 (Fig. 2 and Table 3).

**Table 3:** Predictive diagnostic accuracy of salivary tTG

Best threshold	AUC	Specificity (%)	Sensitivity (%)	Accuracy (%)	NPV (%)	PPV (%)	Precision (%)	FDR (%)
67.97	0.93	98.15	80.00	91.67	96.00	89.83	89.83	10.17

## Discussion

CD is considered a childhood disease, however, up to 20% of adults diagnosed with CD are over 60 years of age [16]. CD manifests itself in a wide range of typical and atypical ways [17–21]. For more than three decades, the CD has been persistently underdiagnosed, and its clinical presentation has changed, leading to difficulties with CD diagnosis [17]. An undetected CD can cause certain complications, such as poor growth, short stature, anemia, neurological issues, liver abnormalities, joint manifestations, dermatitis herpetiformis, osteoporosis, infertility, recurrent aphthous stomatitis, and enamel hypoplasia [22, 23]. The early diagnosis of CD and the treatment with GFD may prevent most of the advanced extraintestinal manifestations. The prognosis for extraintestinal manifestations for children treated with GFD is also better than for adults [23]. Despite the proven benefits of the GFD, it can be exceedingly difficult to completely avoid gluten-containing foods, and adherence to a GFD is estimated to be only 45–80% [24]. The level of GFD adherence in CD patients was not assessed in the present study.

CD, therefore, requires a new diagnostic tool or screening method. In our study, we propose saliva as an alternative to serum serology for the diagnosis of CD. Saliva sampling does not require trained staff, is easy to obtain, prevents accidental exposure to blood, and is inexpensive [25]. In addition, since this method isn't invasive, it is suitable for diagnosing CD in children.

A comparison of salivary and serum levels of tissue transglutaminase IgA was carried out in this study, and the severity of dry mouth in patients with CD was examined. Patients with CD had higher xerostomia rates compared to healthy controls, indicating that CD can adversely affect oral and dental health. The correlation between salivary and serum tTg-IGA was statistically significant for all subjects, and salivary tTg sensitivity and specificity were comparable to previous studies [2, 10]. Several attempts at detecting salivary CD-specific antibodies were made in previous studies.

The results of the present study showed that CD patients had lower mean amounts of amylase in their saliva than the control group, and this difference was statistically significant. Lenander-Lumikari et al., comparing the saliva of CD patients with that of healthy individuals, found that amylase levels were lower in CD patients, confirming the results of this study [26].

The present study did not find a significant difference between CD patients and healthy individuals in terms of other chemical and physical factors, including albumin, total protein, pH, sodium, calcium, potassium, and phosphorus. Lenander-Lumikari et al. reported that albumin and total protein levels were significantly higher in the saliva of CD patients, whereas saliva flow had no significant difference. The results of the study stand in contrast to the present study [26].

According to the results of this study, the mean level of all three interleukins IL-6, IL-18, IL-21 in CD patients was significantly higher than the control group. The study by Manavalan et al. found that the level of tTG -IgA titer correlated statistically with the level of interleukin cytokines in CD. The study by Garrote et al. on CD examined the pathogenesis and cytokines. It found that gluten consumption triggers the production of IL-6, IL-18, and IL-21 interleukins, which can provoke inflammation. This study's findings regarding changes in interleukin levels were in agreement with the present study [27].

## Conclusion

tTG levels in the blood and saliva of CD patients on GFD may be higher than in healthy individuals. Additionally, CD patients should be advised to manage dry mouth and prevent complications associated with it.

## Data Availability

The datasets generated and/or analyzed during the current study are not publicly available due to confidentiality of information but are available from the corresponding author on reasonable request.

## References

[1] Garrote JA, Gómez-González E, Bernardo D, Arranz E, Chirdo F (2008). Celiac disease pathogenesis: the proinflammatory cytokine network. J Pediatr Gastroenterol Nutr.

[2] Bonamico M, Nenna R, Montuori M, Luparia RPL, Turchetti A, Mennini M, Lucantoni F, Masotti D, Magliocca FM, Culasso F (2011). First salivary screening of celiac disease by detection of anti-transglutaminase autoantibody radioimmunoassay in 5000 Italian primary schoolchildren. J Pediatr Gastroenterol Nutr.

[3] Kowalski K, Mulak A, Jasińska M, Paradowski L (2017). Diagnostic challenges in celiac disease. Adv Clin Exp Med Off Organ Wroclaw Med Univ.

[4] Singh P, Arora A, Strand TA, Leffler DA, Catassi C, Green PH, Kelly CP, Ahuja V, Makharia GK (2018). Global prevalence of celiac disease: systematic review and meta-analysis. Clin Gastroenterol Hepatol.

[5] Mohammadibakhsh R, Sohrabi R, Salemi M, Mirghaed MT, Behzadifar M (2017). Celiac disease in Iran: a systematic review and meta-analysis. Electron Phys.

[6] Dahlbom I, Nyberg B-I, Berntson L, Hansson T (2016). Simultaneous detection of IgA and IgG antibodies against tissue transglutaminase: the preferred pre-biopsy test in childhood celiac disease. Scand J Clin Lab Invest.

[7] Dahele AVM, Aldhous MC, Humphreys K, Ghosh S (2001). Serum IgA tissue transglutaminase antibodies in coeliac disease and other gastrointestinal diseases. QJM Int J Med.

[8] Taraghikhah N, Ashtari S, Asri N, Shahbazkhani B, Al-Dulaimi D, Rostami-Nejad M, Rezaei-Tavirani M, Razzaghi MR, Zali MR (2020). An updated overview of spectrum of gluten-related disorders: clinical and diagnostic aspects. BMC Gastroenterol.

[9] Pasinszki T, Krebsz M (2019). Advances in celiac disease testing. Adv Clin Chem.

[10] Bonamico M, Ferri M, Nenna R, Verrienti A, Di Mario U, Tiberti C (2004). Tissue transglutaminase autoantibody detection in human saliva: a powerful method for celiac disease screening. J Pediatr.

[11] Adornetto G, Fabiani L, Volpe G, De Stefano A, Martini S, Nenna R, Lucantoni F, Bonamico M, Tiberti C, Moscone D (2015). An electrochemical immunoassay for the screening of celiac disease in saliva samples. Anal Bioanal Chem.

[12] Husby S, Koletzko S, Korponay-Szabó I, Kurppa K, Mearin ML, Ribes-Koninckx C, Shamir R, Troncone R, Auricchio R, Castillejo G (2020). European society paediatric gastroenterology, hepatology and nutrition guidelines for diagnosing coeliac disease 2020. J Pediatr Gastroenterol Nutr.

[13] Husby S, Murray JA, Katzka DA (2019). AGA clinical practice update on diagnosis and monitoring of celiac disease-changing utility of serology and histologic measures: expert review. Gastroenterology.

[14] van Gils T, Bouma G, Bontkes HJ, Mulder CJ, Brand HS (2017). Self-reported oral health and xerostomia in adult patients with celiac disease versus a comparison group. Oral Surg Oral Med Oral Pathol Oral Radiol.

[15] Thomson WM, Chalmers JM, Spencer AJ, Williams SM (1999). The Xerostomia Inventory: a multi-item approach to measuring dry mouth. Community Dent Health.

[16] Salazar LF, de la Torre FN, Jimenez BV, Colon MN, Hernández JG, Adrados JG (2008). Diagnostic problems in adult celiac disease. Rev Esp Enferm Dig.

[17] Farahmand F, Modaresi V, Najafi M, Khodadad A, Moetamed F, Modarres Z (2011). Prevalence of celiac disease in Iranian children with recurrent abdominal pain referred to a pediatric referral center. Iran J Pediatr.

[18] Trovato CM, Montuori M, Valitutti F, Leter B, Cucchiara S, Oliva S (2019). The challenge of treatment in potential celiac disease. Gastroenterol Res Pract.

[19] Trovato CM, Albanese CV, Leoni S, Celletti I, Valitutti F, Cavallini C, Montuori M, Barbato M, Catalano C, Cucchiara S (2014). Lack of clinical predictors for low mineral density in children with celiac disease. J Pediatr Gastroenterol Nutr.

[20] Persechino F, Galli G, Persechino S, Valitutti F, Zenzeri L, Mauro A, Corleto VD, Parisi P, Ziparo C, Evangelisti M, Quatrale G, Di Nardo G (2021). Skin manifestations and coeliac disease in paediatric population. Nutrients.

[21] Ribaldone DG, Saracco GM, Pellicano R (2018). Pediatric epilepsy and psychiatric comorbidity: could celiac disease diagnosis improve the outcome?. Minerva Pediatr.

[22] Amato M, Zingone F, Caggiano M, Iovino P, Bucci C, Ciacci C (2017). Tooth wear is frequent in adult patients with celiac disease. Nutrients.

[23] Laurikka P, Nurminen S, Kivelä L, Kurppa K (2018). Extraintestinal manifestations of celiac disease: early detection for better long-term outcomes. Nutrients.

[24] Leffler DA, Edwards-George J, Dennis M, Schuppan D, Cook F, Franko DL, Blom-Hoffman J, Kelly CP (2008). Factors that influence adherence to a gluten-free diet in adults with celiac disease. Dig Dis Sci.

[25] Li X, Pomares C, Peyron F, Press CJ, Ramirez R, Geraldine G, Cannavo I, Chapey E, Levigne P, Wallon M (2019). Plasmonic gold chips for the diagnosis of Toxoplasma gondii, CMV, and rubella infections using saliva with serum detection precision. Eur J Clin Microbiol Infect Dis.

[26] Lenander-Lumikari M, Ihalin R, Lähteenoja H (2000). Changes in whole saliva in patients with coeliac disease. Arch Oral Biol.

[27] Manavalan JS, Hernandez L, Shah JG, Konikkara J, Naiyer AJ, Lee AR, Ciaccio E, Minaya MT, Green PH, Bhagat G (2010). Serum cytokine elevations in celiac disease: association with disease presentation. Hum Immunol.

